# Development of quality of care indicators from systematic reviews: the case of hospital delivery

**DOI:** 10.1186/1748-5908-8-42

**Published:** 2013-04-10

**Authors:** Xavier Bonfill, Marta Roqué, Marta Beatriz Aller, Dimelza Osorio, Carles Foradada, Àngels Vives, David Rigau

**Affiliations:** 1 Service of Clinical Epidemiology, Hospital de la Santa Creu i Sant Pau, Sant Pau Biomedical Research Institute (IIB Sant Pau), c/Sant Quintí 89, Barcelona, 08026, Spain; 2 Iberoamerican Cochrane Centre, Hospital de la Santa Creu i Sant Pau, Sant Pau Biomedical Research Institute (IIB Sant Pau), Barcelona, Spain; 3 Department of Paediatrics, Obstetrics and Gynaecology and Preventive Medicine., Universitat Autònoma de Barcelona, Bellaterra, Spain; 4 CIBERESP (CIBER de Epidemiología y Salud Pública), Barcelona, Spain; 5 Research Unit. Consortium for Health Care and Social Services of Catalonia, Barcelona, Spain; 6 Department of Gynaecology and Obstetrics, Corporació Sanitària Parc Taulí, Sabadell, Spain; 7 Department of Gynaecology and Obstetrics, Consorci Sanitari de Terrassa, Terrassa, Spain

**Keywords:** Quality improvement methodologies, Quality indicators, Healthcare, Evidence-based medicine, Obstetrics and gynaecology, Evaluation methodology

## Abstract

**Background:**

The objective of this research is to generate quality of care indicators from systematic reviews to assess the appropriateness of obstetric care in hospitals.

**Methods:**

A search for systematic reviews about hospital obstetric interventions, conducted in The Cochrane Library, clinical evidence and practice guidelines, identified 303 reviews. We selected 48 high-quality evidence reviews, which resulted in strong clinical recommendations using the Grading of Recommendations Assessment, Development and Evaluation (GRADE) system. The 255 remaining reviews were excluded, mainly due to a lack of strong evidence provided by the studies reviewed.

**Results:**

A total of 18 indicators were formulated from these clinical recommendations, on antepartum care (8), care during delivery and postpartum (9), and incomplete miscarriage (1). Authors of the systematic reviews and specialists in obstetrics were consulted to refine the formulation of indicators.

**Conclusions:**

High-quality systematic reviews, whose conclusions clearly claim in favour or against an intervention, can be a source for generating quality indicators of delivery care. To make indicators coherent, the nuances of clinical practice should be considered. Any attempt made to evaluate the extent to which delivery care in hospitals is based on scientific evidence should take the generated indicators into account.

## Background

Quality of care has been defined as the degree to which health services increase the likelihood of desired health outcomes for individuals and populations and are consistent with current professional knowledge [1]. Scientific knowledge is not the only component of the quality of care that must be taken into account, as other structural factors such as process or outcome are also important. In addition, local and particular circumstances of each situation and patients’ preferences cannot be ignored when assessing the appropriateness of a decision [2]. Nevertheless, evaluation of clinical practice through the filter of scientific evidence is an essential enterprise, coherent with the goals of a public health system, the ethical principles of health professionals and the basic rights of citizens, given the possibility that patients might receive inappropriate health care [3,4].

Too little attention has been paid to the correlation between the availability of scientific evidence and clinical practice. A series of studies [5-7] faced the burden of having to analyse *de novo* the evidence relevant to each case. The establishment of *a priori* clinical indicators to be used as performance measures might be more efficient for systematically assessing the degree to which scientific evidence is applied in clinical practice.

Development of indicators based on professional consensus has a long history [8,9], while systematic and explicit methods to incorporate scientific evidence have been developed to a lesser extent. Some recent initiatives, however, followed a systematic approach [10]. Moreover, a successful approach developed by the RAND Corporation combines scientific evidence with professional consensus: the process starts with defining topics of interest, continues with selecting available evidence from different sources, and ends with formulating indicators that are ultimately evaluated by panels of experts through a structured consensus method [11].

In the present study, we restricted the source of indicators of quality of care to systematic reviews (SR), based on the assumption that they provide the highest degree of reliability [12], are increasingly available, and that decision makers increasingly rely on them to cope with the ever-growing volume of healthcare research. Obstetric care during childbirth is particularly suitable for evaluation through evidence-based indicators because it is a field with a relatively high production of SRs. The Cochrane Pregnancy and Childbirth Cochrane Review Group had published hundreds of full SRs, which have been the basis for numerous recommendations and clinical practice guidelines (CPGs) [13]. In addition, some authors have challenged many indicators currently used in obstetrics; a recent study analysed the main indicators currently available (176 in total) and concluded that most did not meet the requirements to measure quality of care [14]. Similar results have been reported in other areas of healthcare [10].

In summary, we generated a set of quality indicators of obstetric care related to childbirth, based on SRs, which could be applicable in different settings and circumstances.

## Methods

The first phase of our project consisted of a literature search and generation of a set of recommendations based on sound evidence, either in favour or against interventions in delivery care; the second phase consisted of developing and validating a set of indicators.

### First phase

Table 1 summarizes the sequential steps followed in the first phase. This mainly consisted of identifying evidence, appraising literature, and generating and grading clinical recommendations. Only SRs of randomized clinical trials were considered.

**Table 1:** First phase: generation of clinical recommendations from systematic reviews

**1. Literature search**	Design and execution of a specific search strategy for identifying systematic reviews (SR).
**2. Selection of SR**	SR were included based on:
	o Field of interest: obstetrics
	o Setting: hospital
	o Relevant to the health topic
	o Intervention of interest: pharmacological or non-pharmacological treatment, under the responsibility of the clinical team and potentially registered at the clinical record or any database.
**3. Appraisal of SR**	An assessment of the methodological quality of each SR; we excluded those documents that did not meet one or more internal validity criteria of SIGN*.
**4. Generation of clinical recommendations (CR)**	Generation of a clinical recommendation (for or against a particular intervention) from each SR. Definitions were provided for population, intervention, comparison and outcomes of interest.
**5. Grading of Recommendations**	Assessment of the quality of evidence and strength of recommendation based on the GRADE† system. Only those recommendations that were considered strong and supported by high quality evidence remained selected.

^*^SIGN: *Scottish Intercollegiate Guidelines Network;* †**GRADE**: *Grading of Recommendations Assessment, Development and Evaluation.*

### Literature search

A literature search was conducted in the Cochrane Database of Systematic Reviews (The Cochrane Library, Issue 3, 2009, and updated in 2011), the Database of Abstracts of Reviews of Effects, and Clinical Evidence to identify SRs assessing obstetric interventions performed in a hospital setting. To retrieve supplementary relevant SR, we consulted the available CPG from the main obstetrical medical societies (the Royal College of Obstetricians and Gynaecologists, the American College of Obstetricians and Gynaecologists) and CPG of obstetric care from main guideline producers (the National Institute for Clinical Excellence (NICE), and the New Zealand Guidelines Group).

### Selection of systematic reviews

Two researchers (MA and DR) independently applied selection criteria to the identified SR: pharmacological or non-pharmacological interventions, under the responsibility of the clinical team and registered at the clinical record or any other database. In case of disagreement, the criterion from a third author (XB) was applied.

### Appraisal of selected reviews

Two researchers (MA and DR) independently appraised each SR and restricted the inclusion to SR that met all internal validity items established by the Scottish Intercollegiate Guidelines Network (SIGN) [15], as assessed on the review’s full text. These criteria assess whether a formulated question is clearly addressed, a description of the methodology is included, the search strategy is sufficiently rigorous, the quality of individual studies is analysed and taken into account, heterogeneity is evaluated and the original authors tried to explain it.

### Generation and grading of recommendations

For each selected SR, we classified the outcomes by relevance (critical, important and relative). Two authors (MA and DR) independently rated the quality of evidence and assessed the strength of recommendations based on the Grading of Recommendations Assessment, Development and Evaluation (GRADE) system [16]. Applicability of the GRADE system to generate quality indicators has been described previously [17,18]. Quality of evidence of critical outcomes was rated high, moderate, low or very low, based on: limitations in design of the primary studies; imprecision, inconsistency and indirectness of the estimates of effects; and likelihood of reporting bias and other biases. A set of clinical recommendations was generated based on balancing the desirable and undesirable consequences of an intervention and the quality of evidence. We used an adaptation of GRADE system, and patients’ values, preferences and resource use were not considered because they are context-specific. Additional file 1: Table S4 shows the modified GRADE system we applied.

Two authors (MA and DR) independently selected recommendations that were considered strong (either in favour or against the application of a given intervention) and based on high quality evidence, at least for the most critical outcomes. In case of disagreements, a third author (XB) was consulted.

### Second phase

#### Development and validation of indicators

From the selected clinical recommendations, we proceeded to construct indicators, following an adaptation of the methods proposed by the American College of Cardiology/American Heart Association (ACC/AHA) [19] and the Agency for Healthcare Research and Quality (AHRQ) [20]. Table 2 shows the general structure of an indicator and the sources of information for each section. Most of the information came either from the SR or current guidelines that summarize both evidence and clinical expertise. Specific sections such as the identification of sources of information to compute the indicator, factors that may explain variability in the results, and specific setting characteristics to ensure the viability of the indicator were needed from clinical experts’ input as well as additional supporting literature.

**Table 2:** General structure of an indicator

**Element**	**Description**	**Source of information**
a. Title	Brief statement of what is to be assessed	Research team
b. Type of Indicator	• Process indicator	Clinical recommendation based on SR
	• Specific indicator of general or medical condition	
	• Indicator of desirable or undesirable events	
	• Indicator based on proportions or means	
c. Definitions	1. Clinical recommendation (PICO format): Clinical situation, population, intervention, comparison and main outcomes	Clinical recommendation based on SR, ICD-9-CM
	• Operational definition of clinical terms in the research question	
	• Definition of contraindications to treatment (if necessary)	
	• Description of the diagnostic and procedure codes ICD-9-CM for the identification of the population	
d. Target population	Definition of the target population	Clinical recommendation based on SR
e. Rationale	• Impact of the clinical condition of interest	SR, CPG
	• Brief description of the selected SR	
	• Summary of the main benefits and/or harms associated with the intervention	
	• Support of the recommendation by main clinical practice guidelines (CPG)	
f. Supporting literature	Main bibliography that supports the indicator (SR and CPG)	SR, CPG
g. Description of indicator population	Operational definition of the indicator (formula)	Clinical recommendation based on SR, clinical experts
	• Numerator / denominator	
	• Exclusion criteria	
h. Sources of information	Description of the sources of information to compute the indicator:	Clinical experts
	• Administrative databases (mainly from inpatient and surgical area)	
	• Clinical documentation (medical history)	
	• Other (survey, etc.)	
i. Standard	Definition of the standard:	Clinical recommendation based on SR
	• Desirable event (↑)	
	• Undesirable event (↓)	
j. Underlying factors	• Factors related to the target population	SR, CPG, Clinical experts
	• Factors related to professionals	
	• Factors related to the hospital	
k. Notes	Other aspects that complement the information summarized by the indicator.	Clinical experts
l. Desired characteristics of a hospital to ensure the viability of the indicator	• Essential features (associated with the identification of the denominator and the numerator)	Clinical experts
	• Desirable features (associated with an acceptable time investment to measure it)	

We subsequently consulted two specialists in obstetrics (CF and AV) to assess our design interpretation of the indicators, and the relevance of the indicators in current practice. This was followed by an email consultation with the authors of the SR on which the indicators were based, asking to what extent they agreed with the formulation of the indicator (content validity, robustness and reliability). The comments received from the review authors or consultants led us to modify or redefine various indicators.

## Results

### Search and selection of systematic reviews

**Figure 1:**
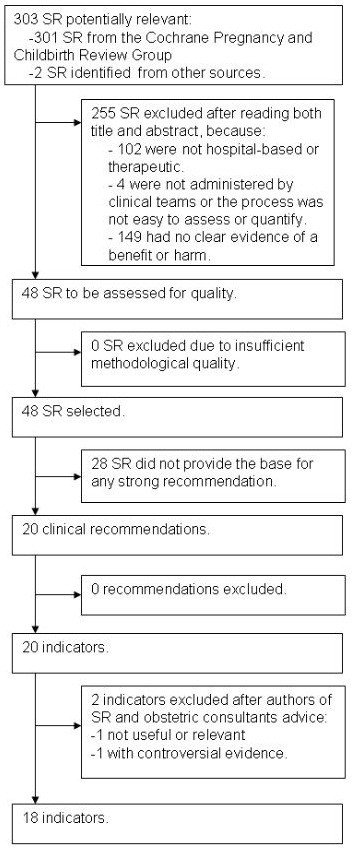
**Short title: Study’s flowchart.** Detailed legend: Flowchart of studies identified in the bibliographic search.

Figure 1 shows the study flowchart. We identified 303 SR, 301 from the search in The Cochrane Library and 2 more [21,22] from the CPG consulted; 102 were excluded for not targeting acute care interventions, 149 for not providing clear evidence (there was no benefit/harm associated with the intervention), and 4 because interventions were not implemented by clinical teams, or the clinical processes were not sufficiently measurable. Then, 48 SR were provisionally selected for further consideration [21-43].

### Quality of evidence assessment and generation of clinical recommendations

The selected reviews consisted exclusively of Cochrane reviews. No SR was excluded based on their quality assessment. In the following stage, 28 SR were excluded for not providing a base for any strong recommendation (either in favour or against an intervention). Thus, we generated 20 clinical recommendations that were both strong and based on high-quality evidence.

### Construction and validation of indicators

Approximately 75% of the authors of the selected SR responded to our request to review the indicator. Overall, they agreed on the indicator proposal, and their comments were used for further improvement of their definition and formulation.

Following advice of the obstetric consultants, two indicators were removed: the proportion of women with singleton pregnancies at risk of preterm delivery to be treated with a combination of corticosteroids with thyrotropin-releasing hormone [33], and the proportion of breech deliveries carried out by caesarean section [32]. The main reasons for excluding these indicators were: the first is an intervention no longer used in clinical practice, and the existing evidence on the second is controversial in nature.

The 18 indicators eventually accepted are shown in Table 3. These indicators are intended to assess the delivery of care during the antepartum period (8 indicators [22-30]), during delivery (8 indicators [21,31,34-40]), at the immediate postpartum (one indicator [41]), and the management of miscarriages (one indicator [42]).

**Table 3:** Quality of care indicators generated in the project

	**Indicator**	**Target population**	**ICD-9 codes**^**a**^	**Indicator formula**^**b**^	**Standard**^**c**^
1	Proportion of women with singleton pregnancies and threatened preterm labour (TPL) who receive corticosteroids^25^	Women with TPL and preterm labour	644.03, 644.10, 644.13, 644.20, 644.21	D: Singleton pregnancies between 26-34w	≈100
				N: Women who received corticoids	
				E: Corticoids contraindications	
2	Proportion of women who are treated with calcium channel blockers (CCB) for inhibiting preterm labour^26^	Women with TPL and preterm labour	644.03, 644.10, 644.13, 644.20, 644.21	D: Pregnancies between 22-34w	≈100
				N: Women who received CCB	
				E: Contraindication to CCB	
3	Proportion of women with threatened preterm labour treated with magnesium sulphate^27^	Women with TPL and preterm labour	644.03, 644.10, 644.13, 644.20, 644.21	D: Women who received pharmacological treatment for TPL	≈0
				N: Women who received magnesium sulphate	
				E: None	
4	Proportion of women with preterm rupture of membranes (PRM) who receive antibiotic treatment^28^	Women with PRM	658.10, 658.11	D: Pregnancies between 22-34w with PRM	≈100
				N: Women who received antibiotics	
				E: None	
5	Proportion of women with post-term pregnancy who give birth after 41 weeks of gestation^29^	Women with >=41w pregnancy	641.X1, 642.X1, 676.X1	D: Women with > =41w pregnancy	≈0
				N: Women with labour induction	
				E: Spontaneous labour, non-urgent caesarean delivery	
6	Proportion of women with severe pre-eclampsia who were treated with magnesium sulphate^30^	Women with severe preeclampsia	642.5	D: Women with severe pre-eclampsia	≈100
				N: Women who received magnesium sulphate	
				E: Contraindication to magnesium sulphate	
7	Proportion of women with eclampsia treated with magnesium sulphate^22,23,24^	Women with eclampsia	642.6	D: Women with eclampsia	≈100
				N: Women who received magnesium sulphate	
				E: Contraindication to magnesium sulphate	
8	Proportion of women with term pregnancies and a breech presentation in which external cephalic version is performed or offered^31^	Women with breech presentation	73.91	D: Breech presentation	≈100
				N: Women in whom cephalic version was performed or offered	
				E: None	
9	Proportion of unjustified episiotomies^34^	Women in whom episiotomy was performed	73.6	D: Women in who episiotomy was performed	≈0
				N: Procedures without any reason documented	
				E: None	
10	Proportion of women whose second-degree perineal tear or episiotomy is repaired with continuous suture^35^	Women with second-degree perineal tear or episiotomy	664.10, 664.11, 644.14, 73.6	D: Women with second-degree perineal tear or episiotomy	≈100
				N: Women in whom continuous suture was performed	
				E: None	
11	Proportion of women who are given an enema during labour^36^	Women in labour	641.X1, 642.X1, 676.X1	D: Women in labour	≈0
				N: Women who were given an enema	
				E: None	
12	Proportion of women having perineal shaving on admission to the delivery room^37^	Women in labour	641.X1, 642.X1, 676.X1	D: Women in labour	≈0
				N: Women for whom perineal shaving was performed	
				E: None	
13	Proportion of women who are administered uterotonics in the third stage of labour^38^	Women in labour	641.X1, 642.X1, 676.X1	D: Women in labour	≈100
				N: Women who received uterotonics	
				E: Contraindication to uterotonics, patient refusal to receive uterotonics	
14	Proportion of women undergoing caesarean section who receive antibiotic therapy^39^	Women on whom caesarean was performed	74.XX	D: Women who received caesarean	≈100
				N: Women who received antibiotics	
				E: None	
15	Proportion of women whose peritoneum is sutured at caesarean delivery^40^	Women on whom caesarean was performed	74.XX	D: Women who received caesarean	≈0
				N: Women for who peritoneum was sutured	
				E: None	
16	Proportion of health professionals who use double gloves when attending a woman with a blood-borne disease^43^	Health professionals performing surgical procedures	None	D: Health professionals who performed surgical procedures in woman with a blood-borne disease	≈100
				N: Health professionals who used double gloves	
				E: None	
17	Proportion of Rh-negative women who are given Anti-D within 72 hours after the birth of an Rh-positive or Rh-undetermined baby^41^	Rh-negative pregnant women	None	D: Rh-negative women with Rh-positive newborn	≈100
				N: Women who received Anti-D	
				E: Women with prior Rh sensitization.	
18	Proportion of women with incomplete miscarriage who, if a surgical evacuation of retained products is carried out, undergo a vacuum aspiration^42^	Women with incomplete miscarriage	634.X1 -638.X1	D: Women with incomplete miscarriage	≈100
				N: Women in who vacuum aspiration was performed	
				E: Contraindication to vacuum aspiration	

^a^ In this column, the value “X” means any number between 0 to 9.

^b^ In this column, (D) Denominator, (N) Numerator (E) Exclusion criteria.

^c^ Theoretical standards: 100% means a desirable event (higher values indicate appropriate performance) and 0% an undesirable event (lower values indicate inappropriate performance).

Indicators are expressed in proportions and refer to process of care, while none refer to structure or outcome. To illustrate the process (see Additional file 2: Table S5) presents the full content of one indicator (proportion of women with singleton pregnancies and threatened preterm labour who receive corticosteroids) and includes an example of its computation.

In 2011, we consulted the Cochrane Library in order to verify the updating status of SR that supports the indicators: all of them have been updated between 2009 and 2011. Three SR changed their conclusions; however none of those changes invalidate the indicators. The first SR, about using antibiotics in women with preterm rupture of membranes, concludes that despite the benefits at short term, during pregnancy, users should be aware of the unknown long term effects on newborns. The second SR, which likewise assesses the use of antibiotics in prophylaxis during caesarean section, provided a similar warning about the unknown long term effects in newborns. The third SR, about active versus expectant management in the third stage of labour, found potential adverse effects with several uterotonics and concludes that information about the benefits and harms should be provided in order to support an informed choice.

## Discussion

The degree of justification or appropriateness of an intervention is directly related to the scientific evidence that supports its implementation and use in practice. Consequently, it seems logical to generate quality indicators through an explicit and systematic process and this has been our purpose. Other recent studies that have developed indicators in a variety of fields, including performance measures [19], clinical practice guidelines [44-46], or a mixed process of evidence appraisal and expert opinion [47,48], have been published. However, to our knowledge, the present study is unique in its focus on SRs.

Several authors warned about potential errors that could be made using quality indicators [49,50]. The most common criticism warns against a construction of quality indicators that is too mechanical. Such a construction would infringe upon the principle that clinical decisions should be flexible in nature, with a lack of individual assessment of each patient and circumstances before applying a particular intervention. Other concerns highlight potential consequences of inflexibility resulting from dichotomizing quality of care into adequate or inadequate in relation to a particular practice, and frequent methodological errors made in the design and construction of indicators [14].

SRs are one of the main instruments for synthesizing available evidence, although they remain little used to generate healthcare explicit quality indicators. In this study, a strategy for the formulation of indicators was based on two basic and differentiated approaches. First, the use of good quality SRs: in this case, predominantly Cochrane reviews as the primary source of evidence to identify interventions for which the potential benefits far outweigh the possible drawbacks; reviews in which that positive balance is not sufficiently significant were excluded. Priority was given to updated secondary sources of literature, so it is unlikely that any subsequent landmark clinical trials for the proposed indicators were missed.

Second, a rigorous and systematic process was conducted to extract relevant data from each review, and the strength of recommendations was assessed by a standardized method (GRADE) [16] to construct each indicator. Only high-quality evidence was considered and this resulted in a strong recommendation (in favour of, or against, the intervention) for the generation of indicators. It implies, according to the GRADE system, that most patients should receive the recommended intervention, or that it can be adopted as a policy in most situations [16]. Moreover, discussions with the obstetric consultants and SR authors resulted in improving additional aspects in the formulation, interpretation and applicability of the indicators. This might be considered a more informal consultation process than other methodologies, such as the aforementioned RAND Corporation approach [11]. Focusing, however, only on highly evidence-based interventions decreases the need to consult experts.

At the end of the process, 18 quality indicators for the delivery of obstetric care in hospitals were identified. Illustrated in Table 2, each proposed indicator has a clear definition, including specific inclusion and exclusion criteria that are consistent with those used in the studies that are the source of evidence, and establishes the population that could benefit from each intervention. All aspects that need to be taken into account for the use of the indicator are described, including clinical situations in which an intervention might not be suitable for a particular patient, meaning that the patient must be excluded from the calculation of the indicator. The possible rejection of the intervention by the patient has also been considered in the formulation of each indicator. This strategy permits one to overcome the classical tension between the generic approach that usually has recommendations contained in a policy document (*e.g*., a clinical guideline) and the necessity of providing personalized care to individuals who are different. In our opinion, only with such an approach can the evaluation of quality of care provided be made, taking the existing evidence and the characteristics and values of each patient simultaneously into account.

The 18 generated indicators represent a conservative sample of the available evidence, since the criteria of consistency, meaningfulness and applicability had priority. We do not expect them to be unique; however, we propose that they should be included in any quality assessment or performance measurement that is made relating to the delivery of care. Since they have been formulated while taking criteria of flexibility and feasibility into account, they could be applied in very different hospital obstetric settings [51].

Some potential limitations of the present study should be noted. First, since indicators help to identify quality problems over time, their applicability and usefulness may depend on the evolving needs of their potential users: policy-makers, health professionals, medical societies, etc., and, theoretically, indicators that specifically address all the issues that are relevant for different stakeholders should be available. However, one characteristic of our methodology is that we have generated indicators based on strong evidence, which should be equally important for all involved parties (*e.g*., assessing that an episiotomy was not performed unless justified). Second, the identified indicators reflect only those aspects of care that are supported by adequate evidence, which do not necessarily cover all the desirable dimensions; however, the reviews represented in the Cochrane Pregnancy and Childbirth Group encompass the most used interventions in the field. Therefore, the results of the present study are very specific (a limited number, if any, of the generated indicators are false positives in relation to their capacity for measuring quality of care) but probably less sensitive (some indicators could be lacking due to the aforementioned limitations) in relation to all possible cases. Third, the rigour applied in our methodology for defining indicators, necessary to guarantee its internal validity, might limit its external validity or applicability in clinical practice. Strictly defining the target population might reduce its applicability, leaving out large groups of people for whom an indicator is not suitable. Finally, calculation of detailed indicators in daily practice might involve the need for accurate information systems and is quite sensitive to the quality of clinical data registration. If clinicians know in advance the criteria applied for calculating quality indicators, they will likely be more aware of the actions that must be considered in each clinical scenario and the necessity of registering them or justifying an alternative. Indicators could not only be included in local clinical guidelines, but could also be part of electronic alarms or clinical reminders to be activated when the hospital information system detects one of the situations labelled as a priority (*e.g*., reminding the administration of antibiotics when a caesarean section is programmed). Future research should concentrate on establishing the corresponding standards for the proposed indicators and interpreting the influence of local circumstances and patient preferences on their observed values.

## Conclusions

The present study demonstrated that the generation of healthcare quality indicators from SRs is feasible and efficient. This is not a simple process, and not all reviews are equally useful in generating indicators. We believe that the thoroughness of the proposed methodology makes these indicators essential references to assess the extent to which the delivery of care is based on scientific evidence. We propose that this methodology be applied to other areas of care where there is sufficiently sound evidence.

## Competing interests

The authors declare that they have no competing interests.

## Authors’ contributions

MA and DR carried out the search selection, data extraction, and evidence assessment. MR and DO helped to methodologically refine the indicators. AV and CF helped to interpret the clinical data and refine the quality indicators. XB conceived the study and participated in its design and coordination. MR, DR and XB drafted the manuscript. All authors read and approved the final manuscript.

## Supplementary Material

Additional file 1: Table S4 Adapted GRADE system for assessing clinical recommendations. Table presenting the adapted GRADE system used in this project to assess clinical recommendations derived from sound systematic reviews. Click here for file

Additional file 2: Table S5 Example of an indicator. Full text of a quality indicator developed in this project and an example of computation based on fictional data. Click here for file
