# Supplementary material for: Development of quality of care indicators from systematic reviews: the case of hospital delivery
Source: Implement Sci. 2013 Apr 10;8:42. doi: 10.1186/1748-5908-8-42 (PMC3626798; doi:10.1186/1748-5908-8-42)
Supplement: Additional file 1: Table S4 — Adapted GRADE system for assessing clinical recommendations. Table presenting the adapted GRADE system used in this project to assess clinical recommendations derived from sound systematic reviews. [file 1748-5908-8-42-S1.doc]

**Table 4. Adapted GRADE system to assess the quality of evidence and grade the strength of recommendations**

| **Assess the quality of evidence:** | Quality from randomized clinical trials is considered initially as "high". Factors that could lower the quality of evidence were: - Limitations in the design and execution of studies - Inconsistent results between studies - No direct evidence - Imprecision of results - Reporting bias - Other relevant sources of bias |
| --- | --- |
| **Grade the strength of recommendations** | Factors considered when moving from evidence to recommendations:  1. Balance between benefits and risks 2. Quality of scientific evidence Neither costs nor values and preferences were taken into account.  Definitions: Strong recommendation:  Beneficial effects outweigh the harms (or vice versa), so most patients should receive the recommended course of action. The recommendation can be adopted as a policy in most situations.  Weak Recommendation: It is not clear if beneficial effects outweigh the harms (or vice versa), so different choices will be appropriate for different patients and clinicians must help each patient to arrive at a decision consistent with his or her values and preferences. Policy making will require substantial debate and involvement of many stakeholders |
| **Representation of the quality of scientific evidence and the strength of recommendations** | Quality of scientific evidence:   - High ⊕⊕⊕⊕ - Moderate ⊕⊕⊕ - Low ⊕⊕ - Very Low ⊕   Strength of recommendations:   - Strong recommendation: **🡩🡩** or **🡫🡫** - Weak recommendation: **🡩?** or **🡫?** |
