# Supplementary material for: Development of quality of care indicators from systematic reviews: the case of hospital delivery
Source: Implement Sci. 2013 Apr 10;8:42. doi: 10.1186/1748-5908-8-42 (PMC3626798; doi:10.1186/1748-5908-8-42)
Supplement: Additional file 2: Table S5 — Example of an indicator. Full text of a quality indicator developed in this project and an example of computation based on fictional data. [file 1748-5908-8-42-S2.doc]

**Table 5. Example of an indicator**

| **1. Title of the indicator**  Proportion of women with singleton pregnancies and threatened preterm labour (TPL) who receive corticosteroids |
| --- |
| **2.** **Characteristics**  Process indicator, specific to the clinical condition, of a desirable event and based on proportions. |
| **3. Definitions**  Definition of indicator: If a woman with a singleton pregnancy is at risk of preterm delivery between 24 and 34 weeks’ gestation, then corticosteroids should be administered, unless contraindicated, because its administration accelerates fetal lung maturation and reduces the risk of: perinatal death, respiratory distress syndrome, cerebroventricular haemorrhage, necrotising enterocolitis, infectious morbidity, need for assisted ventilation and admission to neonatal intensive care units.  Definition of the terms used in the definition of the indicator:  *Threatened preterm labour (ICD-9-CM: Diagnostic code: 644.00, 644.03, 644.10, 644.13, 644.20, 644.21), preterm labour that begins after 22 completed weeks and before 37 completed weeks’ gestation. Target population is limited to those women with a gestation between 24 and 34 completed weeks.  *Contraindications to corticosteroids: systemic infections (such as tuberculosis or chorioamnionitis). Careful assessment of corticosteroid administration in women with severe hypertension or diabetes mellitus is needed. |
| **4. Population**  Women with singleton pregnancies at risk of preterm labour (between 24 and 34 weeks’ gestation). |
| **5. Background**  Preterm birth entails a series of health problems of the newborn resulting from the immaturity of their organs. A systematic review (Roberts 2006, 21 RCTs, 4269 newborn) notes that the administration of one dose of antenatal corticosteroids (betamethasone, dexamethasone or hydrocortisone) reduces neonatal death, respiratory distress syndrome and cerebroventricular haemorrhage in the premature newborn. The treatment does not increase the incidence of maternal death, maternal infection, fetal death, neonatal chronic lung disease or low birthweight. Additionally, it is associated with a reduction in the incidence of neonatal necrotizing enterocolitis and systemic infections in the first 48 hours of life, and a reduced need for respiratory support or admission to the neonatal intensive care unit. The use of antenatal corticosteroids reduces neonatal death even if the delivery occurs within 24 hours after the first dose administration. The quality of evidence is particularly high in women with gestation periods ranging from 24 to 34 completed weeks. In pregnancies over 34 weeks the risk of fetal morbidity and mortality is lower and the benefit associated with treatment is not as clear. There is no evidence to support the use of corticosteroids in multiple pregnancies. Both the Clinical Evidence (Hass 2006) and the WHO Reproductive Health Library (Cuervo 2006) reviews have classified the neonatal corticosteroid intervention as beneficial in women with threatened preterm labour. The clinical practice guidelines identified (RCOG guideline No 7, Crane 2003) also recommended its administration (grade A recommendation). |
| **6. Supporting literature**  -Roberts D, Dalziel S. Antenatal corticosteroids for accelerating fetal lung maturation for women at risk of preterm birth*.* Cochrane Database of Systematic Reviews: Reviews 2006 Issue 3. Chichester, UK: John Wiley & Sons, Ltd. DOI: 10.1002/14651858.CD004454.  -Hass DM. Preterm birth. Clin Evid 2006; 15:1-3.  -Crane J., Armson A., Brunner M., De La Ronde S., Farine D., Keenan-Lindsay L. *et al.* Antenatal corticosteroid therapy for fetal maturation, *J Obstet Gynaecol Can* 2003;25:45-52  -Royal College of Obstetricians and Gynaecologists. Antenatal corticosteroids to prevent respiratory distress syndrome. Royal College of Obstetricians and Gynaecologists; 2004. Guideline No.7.  -Cuervo LG. Intervenciones para prevenir o mejorar el resultado del parto a término o postérmino: Aspectos prácticos de la BSR (última revisión: 6 de Agosto de 2004). Biblioteca de Salud Reproductiva de la OMS, Nº 9, Update Software Ltd, Oxford, 2006. |
| **7. Description of indicator**  Numerator: Women with singleton pregnancies and threatened preterm labour (between 24 and 34 weeks’ gestation) who are given antenatal corticosteroids. Denominator: Women with singleton pregnancies and threatened preterm labour (between 24 and 34 weeks’ gestation).  Exclusion: Women with contraindications to corticosteroids (Absolute contraindications: systemic infections such as tuberculosis or chorioamnionitis). |
| **8. Source of information** Hospitalization and surgical databases, medical history. |
| **9. Standard** Standard: Desirable event (higher values indicate better performance). |
| **10. Factors that may explain the variability of the indicator** Related to women: Women who refuse the administration of corticosteroids. Related to professionals: Difficulties in collecting data. Related to the organization: Clinical practice protocols at the hospital. |
| **11.Notes**  - The corticosteroids of choice must be able to cross the placental barrier. - Administration of corticosteroids should be accepted as routine practice in women with singleton pregnancies and threatened preterm labour (between 24 and 34 weeks’ gestation). - Women should be informed about the importance of the administration of corticosteroids for fetal lung development. - The hospital pharmacotherapeutic guide should discourage the administration of thyrotropin- releasing hormone in combination with corticosteroids in women with threatened preterm labour. |
| **12. Expected characteristics of a hospital to ensure the viability of the indicator** Essential (for identification of the denominator): - Hospital database accessible. - Diagnostic Coding. Desirable (for the complete calculation of the indicator from computerized databases): - Registration of number of weeks’ gestation at admission.  - Computerized record of the treatment administered to women during hospitalization. |
| **13. Example of computation**  As an example, we will compute the indicator using fictional data from an obstetrics unit in a tertiary hospital with 1600 annual deliveries. The target population was identified from a retrospective review of the hospitalization database and medical histories. From the hospital database 37 cases were selected with diagnostic codes corresponding to threatened preterm labour (644.00, 644.03, 644.10, 644.13, 644.20, 644.21). An additional review reduced these cases to a target population of 27 women with a singleton pregnancy between 24 and 34 completed weeks. Review of individual medical histories informed that none of them presented contraindications for corticosteroids. The denominator of the indicator would be the target population of 27 women.  Administration of corticosteroids was confirmed for 23 of these women, which constituted the numerator of the indicator. The quotient between both terms gave a final value for the indicator of 85%. Since no real data is available for comparison with similar hospitals in the area, this result can only be compared to the benchmark of 100% complete appropriateness of care, suggesting that there is ample room for improvement. |
